# Altered Effective Connectivity of Hippocampus-Dependent Episodic Memory Network in mTBI Survivors

**DOI:** 10.1155/2016/6353845

**Published:** 2016-12-15

**Authors:** Hao Yan, Yanqin Feng, Qian Wang

**Affiliations:** ^1^Neuroimaging Laboratory, School of Biomedical Engineering, Shenzhen University Health Science Center, Shenzhen 518060, China; ^2^Key Laboratory of Optoelectronic Devices and Systems of Ministry of Education and Guangdong Province, College of Optoelectronic Engineering, Shenzhen University, Shenzhen 518060, China; ^3^Departments of Linguistics and Psychology, Xidian University, Xi'an 710071, China; ^4^School of Foreign Languages, Northwestern Polytechnical University, Xi'an 710029, China

## Abstract

Traumatic brain injuries (TBIs) are generally recognized to affect episodic memory. However, less is known regarding how external force altered the way functionally connected brain structures of the episodic memory system interact. To address this issue, we adopted an effective connectivity based analysis, namely, multivariate Granger causality approach, to explore causal interactions within the brain network of interest. Results presented that TBI induced increased bilateral and decreased ipsilateral effective connectivity in the episodic memory network in comparison with that of normal controls. Moreover, the left anterior superior temporal gyrus (aSTG, the concept forming hub), left hippocampus (the personal experience binding hub), and left parahippocampal gyrus (the contextual association hub) were no longer network hubs in TBI survivors, who compensated for hippocampal deficits by relying more on the right hippocampus (underlying perceptual memory) and the right medial frontal gyrus (MeFG) in the anterior prefrontal cortex (PFC). We postulated that the overrecruitment of the right anterior PFC caused dysfunction of the strategic component of episodic memory, which caused deteriorating episodic memory in mTBI survivors. Our findings also suggested that the pattern of brain network changes in TBI survivors presented similar functional consequences to normal aging.

## 1. Introduction

Traumatic brain injury (TBI) is a heterogeneous phenomenon with a variety of external force causes, severities, and anatomical injuries. The most common causes of TBI include falls, sports-related injuries, and motor vehicle accidents. TBI severity ranges from mild to severe with brain functional deficits for survivors manifesting across motor, sensory, cognitive, psychological, and socioemotional domains.

The temporal lobes are the most vulnerable areas to acute injury in TBI, in part related to their location near the base of the skull and the free edge of the tentorium [1–3]. Hippocampal atrophy in TBI, which has been demonstrated to be related to injury severity [4], is likely to reflect an aggregated effects of trauma-induced cellular loss that develops over time. Protracted neuronal loss of the hippocampus has been well documented in human postmortem studies [5] and in an extensive experimental animal literature that records cell loss [6–8]. Longitudinal studies showed that hippocampal volumes will decline over a prolonged period from 1 week (largest decline) to 2.5 years [9, 10].

The hippocampus is critical for episodic memory [11, 12], which contains personally experienced events situated in subjective time and space [13]. It has been proposed that both remembering past and imagining novel scenarios rely on an intact hippocampus as the physiologic basis for memory formation and consolidation in a coherent scene [14]. Recent evidence suggests that amnesic patients with hippocampal damage have difficulty not only projecting back in time to mentally simulate the past (retrospection), but also projecting forward in time to mentally simulate novel and specific future scenarios (prospection) [15, 16]. It has also been documented that some TBI survivors seem to live in a timeless world, in a sort of perpetual present experiencing difficulties in traveling back and forward into subjective time [17]. For example, TBI survivors may fail to recall specific events from the personal past [18, 19], may be incompetent in conscious recollection of personal events [19, 20], and may present disturbances in the ability to imagine future (episodic future thinking) [21]. It was putative that the prolonged hippocampal damage may impair the episodic memory system in TBI survivors. However, there was no quantitative MRI study examining how deteriorating structural abnormality of the hippocampus affected episodic memory network in TBI survivors. It prompted us to examine brain connectivity changes of the hippocampus-dependent network in TBI.

Brain connectivity is now being explored by depicting neuronal coupling between brain regions through various techniques [22–24], among which resting state fMRI (rsfMRI) analysis not only has the noninvasive advantage but also possesses additional gains: resting state networks (RSNs) are highly organized in space, reproducible from subject to subject, and allow the search for significant baseline fluctuations to obtain task-free functional network information [25]. To examine cognitive changes specifically, we highlighted how responses in individual brain regions can be effectively combined through functional connectivity. Effective connectivity quantifies directed relationships between brain regions and controls for confounds that limit functional connectivity—features that facilitate insight into functional integration [26]. It overcomes important pitfalls of functional connectivity that limit our understanding of neuronal coupling, such as involvement of functional connection of other cognitive processes, observational noise, or neuronal fluctuations [27]. One popular approach to make effective connectivity analysis is Granger causality analysis (GCA). Coefficient-based GCA is a directed functional connectivity method [28]. It uses multivariate autoregressive models of time series data to illustrate the amount of variance in one region explained by the signal history in another region and quantifies the magnitude and direction of influence of one region time series on another [29]. By examining altered effective interaction in TBI survivors' episodic memory network, we sought to enrich its neural connectivity pathology which is of high importance for accurate diagnosis and early intervention.

The current study planned to focus on mild traumatic brain injury (mTBI) survivors. mTBI is most popular, as it accounts for the overwhelming majority of the head-injured population treated in emergency departments [30]. A recent WHO systematic review suggests that the annual incidence of mild TBI is probably over 600/100,000 [31]. Moreover, moderate and severe TBI consumes most resources per individual, yet mTBI's magnitude and societal ramifications are often underestimated [31]. With the label of “mild,” patients do not seek medical attention, are not systematically assessed, or are lost to medical follow-up [32]. However, many of these injuries may result in long-term difficulties. It has been estimated that 40–80% of mTBI survivors experience postconcussive syndrome, a constellation of physical, cognitive, and behavioral difficulties [33] that may persist up to 2 years after TBI [34]. Along with the significant number of mTBI, these negative outcomes underline the value of accurate identification and adequate management [35]. Further, according to a cohort study with standardized tests of language skills, severe TBI group displayed greater improvement in scores from the acute period to 12 months after TBI. However, scores for the mild-moderate TBI groups remained quite stable over time [36]. To keep subjects more homogeneous and reduce intersubject variabilities, we only focused on patients over 3 months to 14 months after TBI.

## 2. Methods

### 2.1. Participants

Two groups of participants were included in this study. The TBI group was composed of 19 patients (16 males, mean age 38.21 ± 13.42 years) with mild TBI. Screening for mild TBI was based on the World Health Organization's Collaborating Center for Neurotrauma Task Force. Inclusion criteria were (i) conscious survivors at the time of testing; (ii) a favorable outcome (Glasgow Coma Score of 13–15) according to Glasgow outcome scale (GOS); (iii) loss of consciousness (if present) < 30 min, posttraumatic amnesia (if present) < 24 h, and/or other transient neurological abnormalities such as focal signs, seizure, and intracranial lesion not requiring surgery; (iv) an age range of 19–61 years at the time of injury. Exclusion criteria for participants included documented history of neurological disease or long-standing psychiatric condition, preexisting speech and language disorders, drug or alcohol abuse, head injury, and neurological conditions such as brain tumor, stroke, dementia, and Parkinson's. The present study reports data from the 3- and 14-month postinjury assessments. The adult normal control (NC) group consisted of 19 age- and sociocultural level (indexed by the number of years of education) matched healthy controls (13 males, mean age 36.58 ± 7.86 years). All subjects gave written, informed consent after the experimental procedures had been fully explained, and all research procedures were approved by the Ethical Committee of the First Affiliated Hospital of Medical College of Xi'an Jiaotong University and conducted in accordance with the Declaration of Helsinki.

### 2.2. Data Acquisition

The MRI scans were acquired with a 1.5 T MRI scanner (Siemens). A custom-built head holder was used to prevent head movements. Alertness during the scan was confirmed immediately afterward. The MRI protocol involved the high-resolution T1-weighted 3D MPRAGE sequence (echo time (TE) = 2.8 ms, repetition time (TR) = 1900 ms, inversion time (TI) = 1000 ms, flip angle = 8°, slice thickness = 1 mm, field of view (FOV) = 256 mm × 256 mm, and matrix size = 256 × 256), and the single-shot, gradient-recalled echo planar imaging (EPI) sequence with 30 slices covering the whole brain (TR = 2000 ms, TE = 24 ms, flip angle = 90°, FOV = 224 mm × 224 mm, matrix size = 64 × 64, and voxel size = 3.5 mm × 3.5 mm × 4.0 mm). Standard T1, T2, and susceptibility weighted imaging for each patient were examined by two independent neurologists to classify the presence of microbleeds and structural contusion within the clinical group. The scanning datasets were validated visually, and scans were discarded if they did not meet quality control (QC) standards (e.g., regarding artifacts, noise, excessive motion, and missing data). Participants were instructed to stay awake with eyes closed.

### 2.3. Data Processing

Firstly, we adopted functional connectivity analysis to examine brain function and cooperation at a network level by identifying regions that makes up the network of interest. It reflects the degree of signal synchrony between anatomically distant brain regions during resting state. Secondly, we employed multivariate Granger causality analysis (GCA) to quantify the magnitude and direction of influence between network components determined by the functional connectivity analysis step [37].

We used the FMRIB (Oxford Centre for Functional MRI of the Brain, UK) Software Library (FSL) version 6.0 (https://www.fmrib.ox.ac.uk/fsl/) to preprocess raw data and the MATLAB package named REST (resting state fMRI data analysis Toolkit) to make functional connectivity (FC) analysis [38]. The first ten volumes of each functional time course were discarded to allow for steady state stabilization of BOLD fMRI signals. Then, we preprocessed all images firstly through motion correction with MCFLIRT [39] to calculate six head movement parameters, making sure that no participant had head motion with more than 2.0 mm maximum displacement in any direction or 2.0° of any angular motion throughout the course of the scan. Slice timing correction was based on the slice acquisition parameters (slicetimer, FSL), and spatial normalization registered each participant's functional MRI data to its structural MRI data by using Data Processing Assistant for Resting State fMRI (DPARSF), which were then applied to the standard space image MNI-152 atlas (Montreal Neurological Institute, Montreal, QC, Canada) using a 12 parameter affine registration and high-resolution imaging of each subject using 6 parameter affine registrations. REST was then used for linear trend removal and temporal band-pass filtering (0.01, 0.08 Hz) to remove low and high-frequency signal fluctuations. The preprocessed data were spatially smoothed by a Gaussian kernel of 6 mm FWHM (full width at half maximum).

It has been claimed that the left hippocampus represents sequential aspects of episodic experiences and temporal aspects of autobiographical memory, whereas the right hippocampus in humans plays a greater role in spatial processing [40, 41]. The left hippocampus was therefore identified as the seed region of interest (ROI). Our functional connectivity analysis was based on an AAL atlas threshold. For each participant, the mean time course within this ROI was used as the reference time course. A seed functional connectivity analysis was then performed in a whole brain voxel-wise manner with the averaged time courses of the left hippocampus, the white matter, the CSF, and the six head motion parameters as covariates (REST). Individual* r*-maps were normalized to* Z*-maps by using Fisher's* Z* transformation. All Fisher's* Z*-maps were entered into a two-sided one-sample *t*-test to detect regions showing significant functional connectivity with the left hippocampus. Between-group FC differences were determined by two-sample *t*-test detecting the regions showing significantly different FC strength with the left hippocampus.

The whole brain hippocampal network results are reported at *p* < 0.05 (FDR corrected) and with cluster > 10 voxels. The statistics were color-coded and mapped in MNI space, while brain regions were estimated from Talairach and Tournoux after adjustments for differences between MNI and Talariach coordinates with a nonlinear transform. ROIs for further multivariate Granger causality analysis (mGCA) were defined as regions that showed significant functional connectivity with the left hippocampus in the episodic memory network in healthy controls.

The Granger causality is an optimal candidate for its data-driven nature and is widely used in fMRI studies [42]. The entire time series of BOLD signal intensities from ROIs, averaged across voxels within each ROI among subjects of the same group, were normalized to form a single vector per ROI. The mGCA uses directed transfer function (DTF) [43], computed from a multivariate autoregressive model of the time series in the selected ROIs. In this study, we also adopted the weighted DTF with partial coherence to emphasize direct connections and inhibit mediated influences [43, 44]. To assess the significance of path weights, a null distribution was obtained by generating 2500 sets of surrogate data and calculating the DTF [43, 44]. For instance, Fourier transform was applied to each regional time series, and the phase of the transformed signal was randomized. Inverse Fourier transform was then applied to generate one instance of surrogate data. Test statistics were then computed by fitting the VAR model to the surrogate data. In addition, a difference of influence (doi) term was used to assess links that showed a dominant direction of influence to limit potentially spurious links caused by hemodynamic blurring. The doi was compared with the null distribution for a one-tailed test of significance with a *p* value of 0.01 (FDR corrected for multiple comparisons). The stringent threshold was chosen to avoid potentially spurious causal links introduced by low temporal resolution and hemodynamic blurring in the fMRI signal [37].

The high degree nodes were considered to be hubs of network [45]. We calculated “In-degree” (number of Granger causal afferent connections to a node) to find the central targets of network, “Out-degree” (number of Granger causal efferent connections from a node) to find the central sources [46, 47], and “In + Out degree” to find network hubs. Further, three kinds of hubs of the network were defined if the sum of “In-degree,” “Out-degree,” or “In + Out degree” of a node was at least 1 standard deviation (SD) greater than the average degree of all nodes in the network respectively [48, 49]. Between-group degree difference was carried out by the two-sample *t*-test analysis of the “In-degree,” “Out-degree,” and “In + Out degree” of each ROI in each individual, respectively. Connection density difference was determined by calculating between-group difference about the numbers of significantly causal interactions in each individual.

Between-group differences in the causal connectivity graphs were determined using permutation tests to get a data-driven nonparametric approach [50]. Permutation tests constructed a distribution of a test statistic by freely resampling the dDTF values without replacement. The key and only assumption for permutation tests is data exchangeability which means the distributions of two group data are identical under the null hypothesis [51]. Here, we randomly permuted the dDTF values to two new groups. As a result, an empirical distribution of the data sets was constructed using test statistic values for all possible permutations. The true doi obtained with the correct pairs of subjects was then compared with the obtained distribution. Thus, *p* value was calculated by dividing the frequency of permutations presenting more extreme test statistic value by the number of all permutations (10000 times). We consider doi with a *p* ≤ 0.05 as reliable.

## 3. Results

### 3.1. Functional Connectivity Analysis

**Figure 1:**
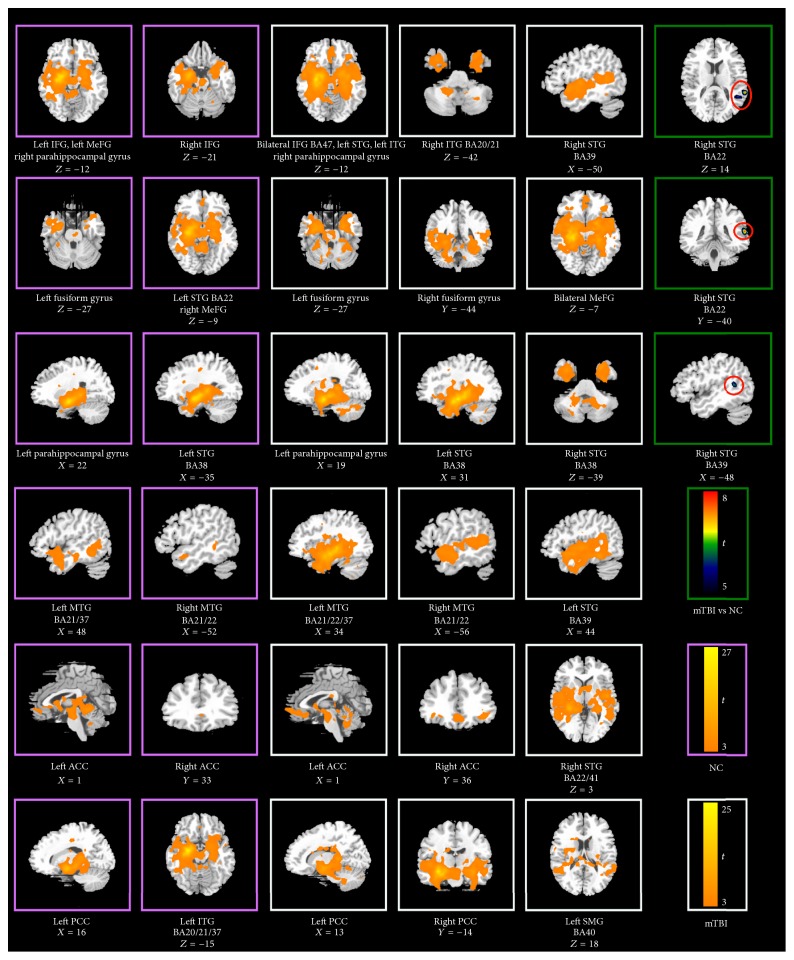
Functional connected hippocampal networks concerning episodic memory in normal controls and mTBI survivors, as well their differences. (i) Covarying brain areas in two groups and their differences were presented in the pink box, white box, and green box, respectively. (ii) BA: Brodmann area; IFG: inferior frontal gyrus; MeFG: medial frontal gyrus; MTG: middle temporal gyrus; ITG: inferior temporal gyrus; STG: superior temporal gyrus; SMG: supramarginal gyrus; ACC: anterior cingulate cortex; PCC: posterior cingulate cortex.

Whole brain functional connectivity maps for the mTBI group, healthy controls, and their contrast results are illustrated in Figure 1 and listed in Table 1. These maps illustrate significant functional connectivity between the left hippocampus and a widespread set of brain regions belonging to the episodic memory system. In common, the hippocampal network of both groups covered bilateral inferior frontal gyrus (IFG, BA47), bilateral medial frontal gyrus (MeFG, BA10/11), left inferior temporal gyrus (ITG, BA21/20), bilateral middle temporal gyrus (MTG, BA 21/22), left fusiform gyrus (BA20/37), bilateral parahippocampal gyrus (PHG), left anterior part of the superior temporal gyrus (STG, BA38), the middle portion of the left STG (BA 41/22), bilateral anterior cingulate cortex (ACC), and left posterior cingulate cortex (PCC). The hippocampus-dependent FC network of the mTBI group additionally involved the bilateral posterior parts of STG (BA39) and left supramarginal gyrus (BA40), as well as the right ITG, right fusiform gyrus, right anterior and middle parts of right STG, and right PCC. By contrast, functional connectivity between the left hippocampus and the right middle/posterior STG (BA22/41 and BA39) were significantly stronger in mTBI survivors than in normal controls. However, functional connectivity between the left hippocampus and other components in the hippocampal network was not significantly stronger in normal controls than that in mTBI survivors. We then determined regions in the hippocampus-dependent functionally connected network in healthy controls as ROIs to form a canonical network and evaluated effective connectivity of the episodic memory network by means of mGCA.

### 3.2. Effective Connectivity Analysis

**Figure 2:**
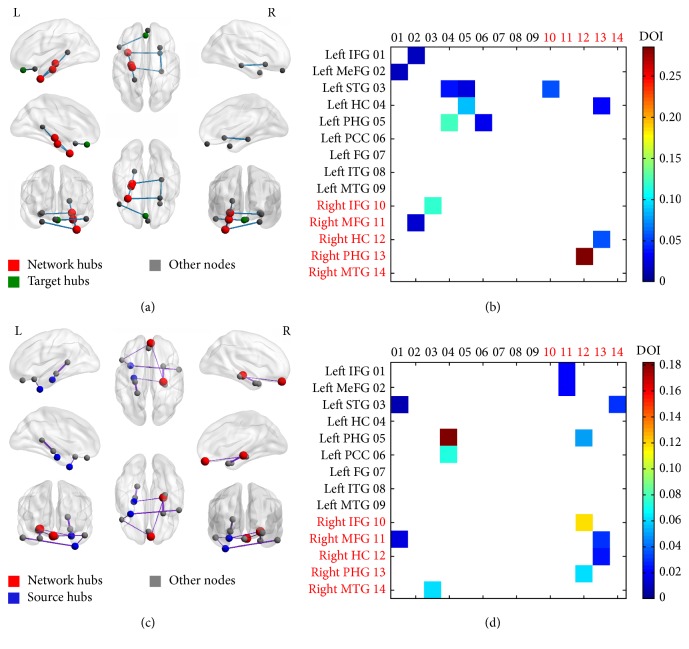
Causal influence of effectively connected episodic memory network in normal controls and mTBI survivors. (i) Connection density in normal controls and mTBI was not significantly different; (ii) significant causal interaction mainly existed between ROIs in the left hemisphere in normal controls, while mTBI survivors presented significant cross-hemisphere connection originating from the right hemisphere; (iii) nodes of source hubs (flow-out hubs), target hubs (flow-in hubs), and network hubs were displayed by different colors, but only the network hub color was presented if their function overlapped (a, c); (iv) significant causal interactions from nodes in *x*-axis to nodes in *y*-axis among all possible causal interactions were presented in (b, d); (v) relative strengths of path weights (in arbitrary units) were indicated by the width of lines; (vi) IFG:inferior frontal gyrus; MeFG: medial frontal gyrus; MTG-middle temporal gyrus; ITG: inferior temporal gyrus; STG-: superior temporal gyrus; FG: fusiform; HC: hippocampus; PHG: parahippocampal gyrus; ACC: anterior cingulate cortex; PCC: posterior cingulate cortex.

A causal connectivity graph was constructed using the thickness of connecting lines to indicate strengths of causal influences (see Figures 2(a) and 2(c)). For both mTBI survivors and healthy controls, causal influences within the episodic memory network presented strongly covarying relations (Figures 2(a) and 2(b)). Results from mGCA analysis showed that causal interactions became denser for the mTBI group than that of the NC group. However, connection density between the two groups was not significantly different from each other (*p* = 0.84). Causal interaction results manifested obvious laterality effect across two groups. For the NC group, significant causal interaction mainly existed between ROIs in the left hemisphere. For example, strong causal outflow originated from the left hippocampus to left anterior STG (aSTG), from the left PHG to left aSTG, and from the left PCC to left PHG. Besides, bidirectional interactions in the left hemisphere between the left hippocampus and left PHG and between the left IFG and left MeFG were significant too. However, we also observed two cross-hemisphere causal connectivity, such as from the left MeFG to right MeFG, from the left aSTG to the right IFG, and from the right PHG to left hippocampus (see Figure 2(b)). For the mTBI group, in the contrary, significant causal interaction mainly existed across hemispheres, mainly flowing out from the right hemisphere. For example, strong causal outflow originated from the right MeFG to left MeFG and from the right hippocampus to left aSTG, as well as bidirectional interactions between the left IFG and right MeFG and between the left aSTG and right MTG. Also significant interhemisphere causal connectivity originating from the left hippocampus to left PHG and left PCC and from the left IFG to left aSTG was detected (see Figure 2(d)). The only overlapped causal interaction in both the mTBI and NC groups was bidirectional connectivity between the right hippocampus and right PHG.

Node degree analysis showed that, in healthy controls, there were three hubs in the hippocampus-dependent episodic memory network, such as the left hippocampus, left PHG, and left aSTG. Specifically, central target hubs (flow-in hubs) were the left MeFG, left hippocampus, and left PHG, while the left PHG and left aSTG were the central source hubs (flow-out hubs). In the mTBI survivors, all three kinds of network hubs shifted from the left to the right hemisphere. Specifically, network hubs were the right MeFG and right hippocampus, the central target hub was the right hippocampus, and central source hubs were the left PHG, left aSTG, and right MeFG. Between-group differences of the “In-degree,” “Out-degree,” and “In + Out degree” values of each ROI were not significantly different (*p* value ranging from 0.09 to 0.96).

**Figure 3:**
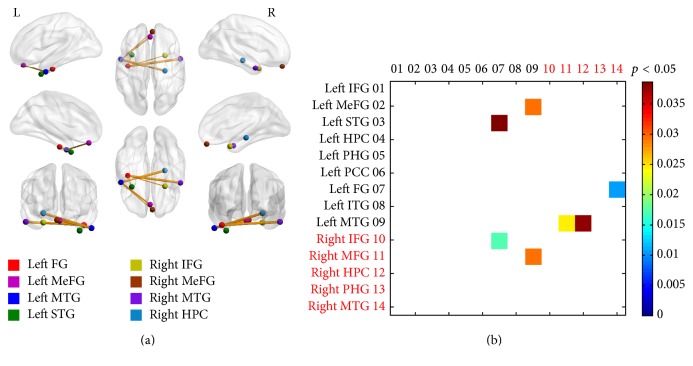
Changes of the driving effect in the episodic memory network between mTBI and normal controls. (i) Most increased driving effects were detected between bilateral structures in patients surviving mTBI. (ii) No decreased causal interaction was found in mTBI survivors. (iii) Relative strengths of path weights (in arbitrary units) were indicated by the width of lines.

Between-group analysis also showed increased driving effect between nodes in bilateral structures (see Figure 3). In detail, increased interhemisphere causal effects were found in the interactions from the left fusiform gyrus to right IFG (NC mean ± SD versus mTBI mean ± SD: 0.0052 ± 0.0061 versus 0.0169 ± 0.0205, *p* = 0.02), from the right MTG to left fusiform gyrus (0.0014 ± 0.0016 versus 0.0044 ± 0.0054, *p* = 0.01), and from the right hippocampus to left MTG (0.0049 ± 0.0110 versus 0.0169 ± 0.0230, *p* = 0.04) and bilateral interaction between the right MeFG and left MTG (0.0030 ± 0.0050 versus 0.0096 ± 0.0114, *p* = 0.02; 0.0031 ± 0.0045 versus 0.0099 ± 0.0205, *p* = 0.01). Increased intrahemisphere causal connectivity was also identified from the left fusiform to the left STG (0.0035 ± 0.0059 versus 0.0113 ± 0.0171, *p* = 0.04) and from the left MTG to left MeFG (0.0036 ± 0.0049 versus 0.0148 ± 0.0263, *p* = 0.03). No decreased driving effect was detected by comparing the mTBI group with that the NC group.

## 4. Discussion

Resting state fMRI maps have been shown to reveal the full distribution of memory-related regions, as they coincide with regions showing activation across a variety of task-based memory studies [52]. The current study sought for covarying areas with the left hippocampus and determined the hippocampus-dependent neural network of episodic memory in mTBI survivors with reference to previous findings. Further effective connectivity analysis of this hippocampal network by GCA revealed significant findings, such as contralateral shift of source, target and network hubs, weakened ipsilateral causal interactions, and increased causal interactions across the two hemispheres, which reflected the neural compensatory mechanism of mTBI survivors.

### 4.1. An Extended Hippocampal Network in mTBI Survivors

The hippocampus-dependent neural network identified in the current study was consistent with previous findings. Specifically, functional connectivity was observed between the hippocampus and many regions of the brain, including the IFG, the MeFG (anterior prefrontal gyrus), the anterior part of STG, the MTG, the ITG, the parahippocampus, and the PCC/retrosplenial cortex [14, 52–55]. These structures are also known to be involved in the default mode network (DMN). As part of episodic memory network [56], the DMN is an interconnected and anatomically defined brain system that preferentially activates in states of relative rest but deactivate during tasks [57]. As expected, mTBI survivors compensated for hippocampal deficits by relying on an intensified and extended functionally connected network. Along with the heavier manipulation of the hippocampal neural network, both the anterior and posterior parts of right STG were overrecruited by mTBI survivors. It was consistent with many neuroimaging studies concerning episodic memory, which have reported that functional brain activity in elders increases in the right hemisphere [58]. Recruitment of the right hemisphere reflected that mTBI patients compensated for decreased functional connectivity of one brain region through recruiting additional brain resources in the contralateral hemisphere to perform a cognitive task. By means of GCA, directed relationships with different linking weights within the episodic memory network provided more information.

### 4.2. A Contralateral Shift of Network Hubs

Causal interaction analysis between ROIs determined by functional connectivity analysis found that hubs of patients' episodic memory network displayed a contralateral shift. In NC, network hubs were the left hippocampus and the PHG (also flow-in hubs, with a dominant role of receiving information) as well as the left aSTG (as a flow-out hub, with a dominant role of sending information). These areas constituted an interactive model of episodic memory in which the anterior STG and hippocampus/PHG were information integration centers for semantic and episodic memory in the declarative memory system.

As a macromodel based on neuropsychological data which presents an interactive construction of memory systems, the MNESIS model (short for Memory NEoStructural Inter-Systemic model) specifies the dynamic and reconstructive nature of memory by highlighting a hierarchical order of three long-term representation systems of perceptual memory (the lowest level), semantic memory, and episodic memory (the highest level) and adds two retroactive processes of memory semanticization and perceptual memory transfer during experience reliving [59]. The perceptual representation system receives, stores, and makes the basic unit of information about perceptual features of physical objects available to other systems [60]. Semantic memory refers to one's noetic awareness of the existence of the world and objects, events and other regularities in it, independent of self, autonoetic awareness and time [60]. When semantic information of the memory, which has no contextual richness but present a schematic version of the memory, is established, retrieval processes are required to reactivate its mental representations and return the individual to his or her conscious experience of the event [61]. These personal experiences covered detailed contextual information [62], scene construction [63], and a sense of reliving or autonoetic consciousness [64].

Semantic memory encompasses a rich fund of general knowledge about the world, represented in visual, olfactory, gustatory, tactile, and auditory cortices [65, 66], conducive to the identity of perceptual events. It is proposed that these multiple sensory inputs are converged in the left anterior temporal lobe (ATL), a transmodal representational hub [65], to form a concept. The ATL as a concept formation center in the semantic memory network has been demonstrated by magnetoencephalography, distortion-corrected functional MRI, PET, or repetitive transcranial magnetic stimulation techniques [67]. Meanwhile, the hippocampus is always involved whenever detailed; contextual information is recalled according to the Transformation Hypothesis of memory consolidation [68] and the Binding of Items and Contexts model [69]. Personal experience is represented as a pattern of features that correspond to different facets processed during the encoding of the episode [70]. The hippocampus is more adept at associating multiple attributes of differential forms of memory than other structures [71]. Inputs from regions of the recognition (or context) memory network are not directly involved in memory strength, but converge at the hippocampus to become cohesive memories of individual events via the formation (“binding”) of episodic memory [72, 73]. After binding, the outputs of the hippocampus return to cortical regions from which the inputs arose. Thus, the hippocampus performs complex high-resolution binding of the different qualitative aspects of an event, both at encoding and at retrieval [58]. The parahippocampal area is also an important hub as it enables the communication between the hippocampus and neocortical areas [74]. It has been proposed that the PHG provides contextual information about the “where” and “when” of a target item for memory encoding to the hippocampus to bind new memories and link the memory of that particular episode within a larger network [11, 59, 69], underlining its integrative and maintenance functions.

In mTBI survivors, both the left hippocampus/PHG and anterior STG were no longer network hubs. It indicated that external forces can severely damage human declarative memory. These network hubs in normal controls were replaced by the right hippocampus and right MeFG. It was compatible with the pathological observation that reduced volume of the temporal lobe is commonly found following moderate to severe TBI, due to abrasive contact with bony plates of the skull during the acute injury. The observed compensation mechanism in mTBI was also consistent with previous findings. A latest research, which performs a direct test of the relationship between episodic memory's perceptual richness and hippocampal function, claims that the right hippocampus plays an important role in the recruitment of posterior cortical regions that support the representation of perceptual memory content [75]. In the current study, mTBI survivors enhanced effective connectivity of the right hippocampus with other regions. It reflected that mTBI survivors compensated for episodic memory dysfunction by means of overenrollment of the perceptual memory system.

Meanwhile, greater activation of the right PFC also indicated an optimized compensation mechanism in the mTBI survivors. The PFC is generally thought to be the primary site of scaffolds to compensate for declines in functioning in other regions, due to its versatile and flexible nature [76]. It was suggested that at least some of the age-related overrecruitment in prefrontal cortex may reflect attempted compensation for reduced activation in the hippocampus [77]. A recent review also highlighted that the PFC was heavily activated during memory encoding and retrieval in elderly participants [78]. With reference to neurocognitive models, it was postulated that the strategic component of episodic memory depends primarily on the PFC to constrain memory search and monitor the appropriateness of recovered memories [79]. The intensified interconnection between the right MeFG and other regions in the current study showed that mTBI survivors manipulated the right MeFG to a greater degree to make up for the left hippocampus deficit. We attributed its greater involvement to increased strategic retrieval/recombination demands. However, overactivation of the PFC is not always related to better performance but a poorer memory [80], because recruitment of additional brain regions might come with additional cost [81]. We postulated that overrecruitment of the right MeFG may cause dysfunction of its primary role due to overload. The affected executive control and working memory thus led to failed episodic memory retrieval.

A study analyzing story narratives from 10 participants with TBI reported a normal microlinguistic processing (lexical and syntactic) but impaired macrolinguistic abilities (pragmatic, cohesive, and coherent) in TBI survivors [82]. The authors defined these patients as nonaphasic and suggested that their confused and impoverished language was caused by reduced ability to organize information at the macrolinguistic level (unable to guide comprehension and production of logical relationships, both temporal and causal, between agents and events) [83]. Our primary finding of a disrupted causal connectivity between the left aSTG (the concept formation center in the semantic memory network) and the hippocampus may provide their research a possible explanation. We demonstrated that it was due to the broken-down interaction of the concept forming center and the context binding center that caused the abnormal macrolinguistic processing but preserved lexical and syntactic competence in TBI survivors. Hence, TBI survivors always presented a confused and impoverished language but no obvious symptoms of other language deficits, such as anomia.

## 5. Conclusion

Brain injury of the hippocampus was consistently reported to cause a chronic post-TBI episodic memory impairment. By examining altered effective connectivity of the episodic memory network in mTBI survivors, we found that the pattern of brain network changes detected in TBI survivors has similar functional consequences to normal aging [84]. Even though functionally connected regions with the hippocampus were extended, dysfunction of neural network hubs of the aSTG, hippocampus, and PHG in the dominant hemisphere and overrecruitment of the right MeFG lead to an abnormal episodic memory network. Our findings also demonstrated that effective connectivity analysis was more suitable to represent the working mechanism of an episodic memory network than functional connectivity analysis. To the best of our knowledge, this is the first in vivo demonstration of the dynamic relationships between nodes in the hippocampal episodic memory network in mTBI survivors.

## Figures and Tables

**Table 1:** Functionally connected brain areas with the left hippocampus in normal controls and mTBI survivors (voxel level: *p* < 0.05, FDR corrected).

		Normal controls					mTBI					mTBI versus normal controls				
		Talairach			*t*	*V*	Talairach			*t*	*V*	Talairach			*t*	*V*
		*x*	*y*	*z*	value	voxels	*x*	*y*	*z*	value	voxels	*x*	*y*	*z*	value	voxels
Frontal cortex																
Inferior frontal gyrus BA47																
															
L		−45	17	−11	5.00	37	−42	14	−11	5.74	82					
R		24	8	−18	4.48	84	33	29	−12	8.25	123					
Medial frontal gyrus BA10/11																
															
L		0	37	−12	4.35	20	0	49	−8	6.55	103					
R		3	46	−10	4.09	18	3	49	−8	6.69	65					
Temporal cortex																
Inferior temporal gyrus BA21/20																
															
L		−50	−50	−10	6.08	72	−56	−9	−12	5.97	135					
R							36	−2	−35	5.00	73					
Middle temporal gyrus BA 21/22																
															
L		−50	2	−25	5.51	300	−36	1	−33	7.35	476					
R		50	2	−18	4.00	20	53	5	−10	6.24	383					
Fusiform gyrus BA20/37																
															
L		−36	−10	−22	5.56	167	−36	−13	−22	8.39	246					
R							42	−44	−15	6.48	182					
Parahippocampal gyrus																
															
L		−24	−12	−15	26.91	518	−21	−12	−15	23.79	628					
R		27	−24	−9	8.75	193	24	−1	10	11.00	477					
Hippocampus																
R		30	−24	−9	7.14	130	36	−9	−12	10.72	183					
Superior temporal gyrus BA38																
															
L		−30	10	−31	5.72	181	−33	−1	−13	8.76	255					
R							27	4	−33	8.66	284					
Superior temporal gyrus BA22/41																
															
L		−42	−27	−6	4.60	34	−53	−9	−10	7.13	439					
R							48	−11	3	8.14	417	62	−40	13	8.18	51
Superior temporal gyrus BA39																
															
L							−45	−52	16	5.17	37					
R							48	−52	11	6.62	39	48	−52	11	6.13	17
Parietal cortex																
Supramarginal gyrus BA40																
															
L							−56	−49	19	4.60	13					
R																
Subcortical cortex																
ACC																
															
L		−3	0	−8	5.93	40	−3	3	−3	4.34	41					
R		3	32	−7	3.45	23	3	34	−9	4.00	79					
PCC																
															
L		−18	−40	10	5.38	21	−15	−43	8	5.12	48					
R							12	−34	18	4.24	28					

BA: Brodmann area; ACC: anterior cingulate cortex; PCC: posterior cingulate cortex.
